# Curvature model for nanoparticle size effects on peptide fibril stability and molecular dynamics simulation data

**DOI:** 10.1016/j.dib.2022.108598

**Published:** 2022-09-14

**Authors:** Torsten John, Lisandra L. Martin, Herre Jelger Risselada, Bernd Abel

**Affiliations:** a Leibniz Institute of Surface Engineering (IOM), Permoserstraße 15, 04318 Leipzig, Germany; b Wilhelm-Ostwald-Institute for Physical and Theoretical Chemistry, Leipzig University, Linnéstraße 3, 04103 Leipzig, Germany; c School of Chemistry, Monash University, Clayton, Victoria 3800, Australia; d Institute for Theoretical Physics, Georg-August-Universität Göttingen, Friedrich-Hund-Platz 1, 37077 Göttingen, Germany

**Keywords:** Aggregation, Self-assembly, Nanoparticle, Size, Curvature, Oligomer, Peptide, Amyloid, Aß_40_, amyloid beta (1–40), MD, molecular dynamics, PBC, periodic boundary conditions, PDB, protein data bank, SPC, simple point charge

## Abstract

Nanostructured surfaces are widespread in nature and are being further developed in materials science. This makes them highly relevant for biomolecules, such as peptides. In this data article, we present a curvature model and molecular dynamics (MD) simulation data on the influence of nanoparticle size on the stability of amyloid peptide fibrils related to our research article entitled “Mechanistic insights into the size-dependent effects of nanoparticles on inhibiting and accelerating amyloid fibril formation” (John et al., 2022) [1]. We provide the code to perform MD simulations in GROMACS 4.5.7 software of arbitrarily chosen biomolecule oligomers adsorbed on a curved surface of chosen nanoparticle size. We also provide the simulation parameters and data for peptide oligomers of Aß_40_, NNFGAIL, GNNQQNY, and VQIYVK. The data provided allows researchers to further analyze our MD simulations and the curvature model allows for a better understanding of oligomeric structures on surfaces.

## Graphical Abstract

**Figure:**
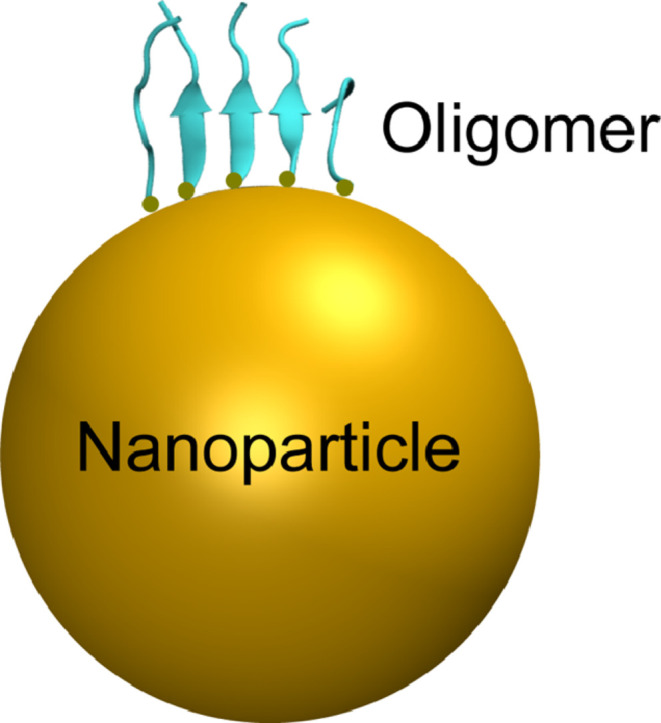

**Specifications Table**

**Table:** 

Subject	Chemistry
Specific subject area	Theoretical modelling of oligomer and fibril stability on curved surfaces
Type of data	Figure Simulation code (.c) Simulation data (.top, .itp, .gro, .ndx, .mdp, .edr, .tpr, .trr)
How the data were acquired	MD simulations were performed in the GROMACS 4.5.7 software. The simulations were run on a high-performance computing cluster.
Data format	Raw
Description of data collection	Peptide oligomers consisting of 5 (5mer) or 10 (10mer) monomers of Aß_40_, NNFGAIL, GNNQQNY and VQIVYK were constructed using the fibril structure information obtained from the Protein Data Bank (PDB) (Aß_40_ (ID: 2M4J) [2], NNFGAIL (ID: 3DGJ) [3], GNNQQNY (ID: 2OMM) [4] and VQIVYK (ID: 2ON9) [4]). The structural stability of these preformed oligomers on curved surfaces was investigated using a spherical harmonic potential. To apply this, the *N*-terminus of the central monomer of the peptide oligomers was positioned in the center of the simulation box. We implemented a curvature model in the GROMACS 4.5.7 code [5], [6], [7], [8], [9]. This allows the specification of the nanoparticle radius, the index group of atoms to be constrained to a spherical harmonic potential, and the force constant of the potential, a measure of how strongly the index group atoms are attracted to the potential surface (*E(r-r_0_)* = 1/2·*k*·(*r-r_0_*)^2^, where *k* is a force constant, *r_0_* is the effective radius of the nanoparticle, and *r* is the position of the index group with respect to the nanoparticle center). The *N*-terminus of the peptides was constrained to be attracted by the curvature potential. The overall peptide experienced the excluded volume imposed by the nanoparticle *via* a flat bottom potential, *E(r-r_0_)* = 1/2·*k*·(*r-r_0_*)^2^ if *r < r_0_* and *E(r)* = 0 otherwise. We note that the latest versions of GROMACS allow the use of spherical flat bottom potentials, which in principle allow a similar simulation setup. The peptide oligomers were solvated in explicit water (Simple Point Charge, SPC) [10] and 150 mM sodium chloride was added as a physiological salt and to electroneutralize the systems. Each peptide oligomer was simulated in triplicate at 300 K for 100 ns in GROMACS 4.5.7, each with independent initial velocities. Each system was energy minimized and equilibrated before running the 100 ns simulations. The selected simulation parameters are provided (.mdp files).
Data source location	Institution: Leibniz Institute of Surface Engineering (IOM) City: Leipzig Country: Germany
Data accessibility	Repository name: Zenodo Data identification number: 10.5281/zenodo.6412773 Direct URL to data: https://doi.org/10.5281/zenodo.6412773
Related research article	T. John, J. Adler, C. Elsner, J. Petzold, M. Krueger, L.L. Martin, D. Huster, H.J. Risselada, B. Abel, Mechanistic insights into the size-dependent effects of nanoparticles on inhibiting and accelerating amyloid fibril formation, J. Colloid Interface Sci. 622 (2022) 804–818. https://doi.org/10.1016/j.jcis.2022.04.134

## Value of the Data

• The curvature model and molecular dynamics (MD) simulation data provided in this article allow researchers to understand the stability of amyloid peptide oligomers on curved surfaces. Amyloid peptides are linked to neurodegenerative diseases and therefore knowledge of the mechanistic influence of nanoparticles on fibrillation kinetics is relevant [11].
• Our data allow researchers to visualize the simulations and analyze them in further directions. Researchers can also vary the parameters, i.e. the nanoparticle radius and the potential force constant, to extend our simulations under different conditions.
• The curvature potential that we implemented in GROMACS 4.5.7 can be used directly by other researchers for any molecule or oligomer arranged on a curved surface. This can be useful for a variety of applications.

## 1 Data Description

**Fig. 1:**
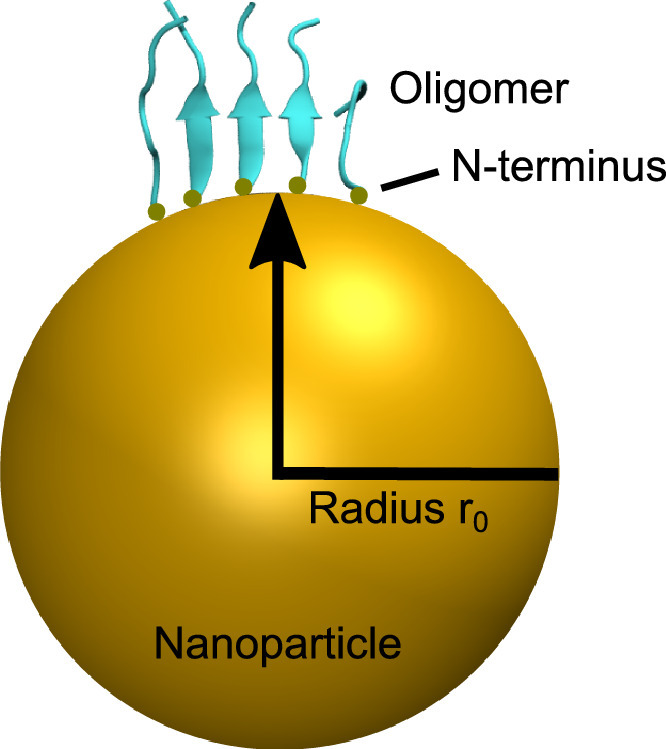
Schematic representation of the nanoparticle curvature model. The nanoparticle surface is modelled implicitly. The nanoparticle radius *r_0_* and the force constant of the potential *k* can be varied. In our case, the *N*-terminus of the peptides was adsorbed onto the nanoparticle surface using a spherical harmonic potential. The *N*-terminus experiences an attractive potential towards the surface, while the overall peptide experiences repulsion once it reaches the surface (excluded volume, *r* < *r_0_*).

The data published in this article includes a modified code file that can be implemented into GROMACS 4.5.7 software to use our curvature model. In addition, molecular dynamics (MD) simulation data is provided and detailed below. The interpretation of the data and results was published in the Journal of Colloid and Interface Science [1]. The curvature model uses a spherical harmonic potential to implicitly describe a nanoparticle of radius *r_0_*, while the attraction of the adsorbed oligomer is controlled by defining the potential force constant *k* (see Fig. 1). A defined subgroup of atoms, in our case the peptide *N*-terminus is constrained *via* the potential; the rest of the oligomer experiences repulsion once it reaches the surface.

### 1.1 Modified md.c code for GROMACS 4.5.7 (Modified-md.c-file-GROMACS-457.zip)

The modified md.c file is used to install a modified GROMACS 4.5.7 version on the computer. This should be a separate installation.

1) Download GROMACS 4.5.7 from the software website (https://ftp.gromacs.org/pub/gromacs/).
2) Extract the gromacs-4.5.7.tar.gz archive.
3) Go to /src/kernel/, and replace the md.c file.
4) Go back into highest gromacs-4.5.7 directory. Create new directory (mkdir exec) and change there (cd exec)
5) Run the command: cmake

We point out that the latest GROMACS versions allow the use of spherical flat bottom potentials, which in principle allow a similar simulation setup as our in-house modification.

### 1.2 MD simulation data (Ab40.zip, NNFGAIL.zip, GNNQQNY.zip, VQIVYK.zip, forcefield.zip)

The MD simulation files for 5mer and 10mer of Aß_40_, NNFGAIL, GNNQQNY, and VQIVYK peptides are provided. Researchers can visualize our simulations in their favorite software and use our files to set up their own systems.

### 1.3 Directory Structure for Ab40, NNFGAIL, GNNQQNY, VQIVYK

**Table:** 

<Peptide Name>/ <Oligomer Size>/1_top	Topology files (*.top, *.itp), Starting structure (*.gro) and index file (*.ndx)
/2_em	Energy minimization files (*.mdp, *.gro, *.edr, *.tpr, *.trr)
/<Radius>/3_equi	Equilibration files (*.mdp, *.gro, *.edr, *.tpr, *.trr)
/<4_Run-x>	Simulation files (*.mdp, *.gro, *.edr, *.tpr, *.trr)

**Table:** 

Options	<Peptide Name>	Ab40, NNFGAIL, GNNQQNY, VQIVYK
	<Oligomer Size>	5mer, 10mer
	<Radius>	5nm, 20nm

### 1.4 Submission File Typess

The same file types are provided for each peptide, oligomer size, and nanoparticle radius.

**Table:** 

*1_top Folder: This folder contains the topology, initial structure and index files.*	
*.top	This is the GROMACS topology file. It contains information about the system and the used force field. It is read by grompp to create a binary topology (*.tpr).
*.itp	These are GROMACS topology files that are included in the *.top file (info for the peptides).
*.gro	This is the initial GROMACS structure file. It can be visualized in sofware, such as VMD [12].
*.ndx	This is the GROMACS index file. It contains user definable sets of atoms as groups. Two groups must be assigned for the curvature model: The groups “NANO” and “PEPTIDE” are assigned the atoms that are bound to the attractive potential (“NANO”) and the entire bound molecules (“PEPTIDE”), i.e. the peptide *N*-termini and all peptide atoms.
*2_em Folder: This folder contains the files for the energy minimization.*	
*.edr	This is the GROMACS energy output file with information about e.g. energies, temperature and pressure.
*.gro	This is the GROMACS structure file of the last frame.
*.tpr	This is the GROMACS portable binary run input file.
*.trr	This is the GROMACS trajectory file, including information on coordinates, velocities, forces and energies.
*.mdp	This is the GROMACS parameter file.
*3_equi Folder: This folder contains the files for the equilibration.*	
*4_run-x Folder: This folder contains the files for the production simulations.*	
The file types for the 3_equi and 4_run-x folders are similar to the 2_em Folder.	

The *.gro and *.trr files can be used to visualize the structures in visualization software such as VMD [12]. The *.mdp and *.top files are useful for repeating or extending the simulations and can serve as a guide to create different simulations with our curvature model. We also provide a force field folder for the OPLA/AA force field that we used for the peptides in the related research article [1] and the data included here [13,14]. We note that any other force field can be used in combination with the curvature potential.

## 2 Experimental Design, Materials and Methods

The development of the curvature model and the method for the molecular dynamics (MD) simulations were published in the related original research article [1]. Detailed practical information on using the data and setting up simulations can be found here. The stability of peptide oligomers was studied using external potentials implemented in the GROMACS 4.5.7 software [5], [6], [7], [8], [9]. The surface of the implicit nanoparticle was designed to be right in the center of the simulation box.

### 2.1 Preparation of Peptide Oligomers for Simulation

To prepare the peptide oligomer structures for the MD simulations, the peptide *N*-termini were centered in the simulation box. The peptide fibril was oriented perpendicular to the *yz* plane, i.e. the peptides were oriented along the *x*-axis. The center of the nanoparticles is in *x* = box center (box size in *x*/2) – *r*_*0*_, *y* = box center (box size in *y*/2), *z* = box center (box size in *z*/2). The simulation box was chosen large enough to ensure that the peptides do not cross the periodic boundary conditions during the simulation (by no means in the *x*-dimension) (2 nm distance between solute and box edges). The nanoparticle itself was not subjected to periodic boundary conditions (PBC). Index groups for the peptide *N*-termini (NANO) and the whole peptides (PEPTIDE) were created. The *N*-termini (NANO group) experience an attractive potential while the entire peptide (PEPTIDE group) experiences repulsion within the excluded volume of the implicit nanoparticle.

Attractive spherical harmonic potential: *E(r-r_0_)* = 1/2·*k*·(*r-r_0_*)^2^

where *k* is the force constant (500 kJ nm^−2^ mol^−1^), *r_0_* is the effective radius of the nanoparticle, and *r* is the position of the *N*-terminal heavy atom in relation to the nanoparticle center

Flat bottom potential for excluded nanoparticle volume: *E(r-r_0_)* = 1/2·*k*·(*r-r_0_*)^2^ if *r < r_0_* and *E(r)* = 0 otherwise.

### 2.2 Parameters for MD Simulations

The following parameters were introduced in the *.mdp GROMACS files and are used to adjust the curvature potential:

**Table:** 

user1-grps = NANO
; Index group for the atoms that are to experience the attractive potential, in our case the*N*-terminal nitrogen atoms of the peptides. These must be placed within the center of the box.
user2-grps = PEPTIDE
; Group containing all peptide atoms, they experience repulsive interactions.
userint1 = 1; on switch of the module
userint2 = 0
; Center of nanoparticle is 0.5*BOX X - Radius,0.5*BOX Y,0.5*BOX Z.
userint3 = 0
userint4 = 0
userreal1 = 2.5; Radius of Nanoparticle in nm
userreal2 = 500; Force constant in kJ nm^−2^mol^−1^
userreal3 = 0

The ‘userreal1’ and ‘userreal2’ parameters can be varied for different nanoparticle size and surface attraction of the oligomer.

### 2.3 Commands for Running MD Simulations

The commands for running the energy minimization, equilibration, and production runs are shown below as examples:

**Table:** 

*Energy minimization:*
grompp -f Ab40-em.mdp -n ../1_top/Ab40_5mer_solv_ions.ndx -c ../1_top/Ab40_5mer_solv_ions.gro -p ../1_top/Ab40-5mer.top -o Ab40_5mer_em.tpr
mdrun -s Ab40_5mer_em.tpr -c Ab40_5mer_em.gro -deffnm Ab40_5mer_em
*Equilibration:*
grompp -f Ab40-5nm-equi.mdp -n ../../1_top/Ab40_5mer_solv_ions.ndx -p ../../1_top/Ab40-5mer.top -c ../../2_em/Ab40_5mer_em-2.gro -o Ab40_5mer-5nm_equi.tpr
mdrun -s Ab40_5mer-5nm_equi.tpr -c Ab40_5mer-5nm_equi.gro -deffnm Ab40_5mer-5nm_equi
*Production run:*
grompp -f md_curvature-5nm-100ns-1.mdp -n ../../1_top/Ab40_5mer_solv_ions.ndx -p ../../1_top/Ab40-5mer.top -c ../3_equi/Ab40_5mer-5nm_equi.gro -o Ab40_5mer-5nm-100ns-1.tpr
mdrun -s Ab40_5mer-5nm-100ns-1.tpr -c Ab40_5mer-5nm-100ns-1.gro -deffnm Ab40_5mer-5nm-100ns-1

### 2.4 Visualizing Implicit Nanoparticles in VMD

To visualize the implicit nanoparticles in the VMD visualization software, the following TkConsole commands can be used.

**Table:** 

Choosing the color of the sphere
draw color 32
Deleting all drawings
draw delete all
Drawing nanoparticle sphere for Aß_40_, 10mer (1st line: 5 nm radius, 2nd line: 20 nm radius)
draw sphere {53.55 49.24 44.015} radius 25 resolution 50
draw sphere {-21.45 49.24 44.015} radius 100 resolution 100
Drawing nanoparticle sphere for NNFGAIL, 10mer
draw sphere {4.365 45.515 28.595} radius 25 resolution 50
draw sphere {-70.635 45.515 28.595} radius 100 resolution 100
Drawing nanoparticle sphere for GNNQQNY, 10mer
draw sphere {5.88 45.285 26.545} radius 25 resolution 50
draw sphere {-69.12 45.285 26.545} radius 100 resolution 100
Drawing nanoparticle sphere for VQIVYK, 10mer
draw sphere {2.93 43.885 29.12} radius 25 resolution 50
draw sphere {-72.07 43.885 29.12} radius 100 resolution 100
Drawing nanoparticle sphere for Aß_40_, 5mer
draw sphere {53.55 36.01 44.015} radius 25 resolution 50
draw sphere {-21.45 36.01 44.015} radius 100 resolution 100
Drawing nanoparticle sphere for NNFGAIL, 5mer
draw sphere {4.365 33.275 28.595} radius 25 resolution 50
draw sphere {-70.635 33.275 28.595} radius 100 resolution 100
Drawing nanoparticle sphere for GNNQQNY, 5mer
draw sphere {5.88 32.95 26.54} radius 25 resolution 50
draw sphere {-69.12 32.95 26.54} radius 100 resolution 100
Drawing nanoparticle sphere for VQIVYK, 5mer
draw sphere {2.93 31.725 29.12} radius 25 resolution 50
draw sphere {-72.07 31.725 29.12} radius 100 resolution 100

MD simulations were performed for four peptides with two different oligomer sizes at two different curvatures using a constant force constant (i.e. binding affinity). Our model allows flexibility for different peptides, oligomer sizes, curvatures, and binding affinities.

## Ethics Statements

The authors declare that the work does not involve the use of human subjects, animal experiments, or data collected from social media platforms, being exempt from an ethic approval process.

## CRediT authorship contribution statement

**Torsten John:** Conceptualization, Methodology, Software, Validation, Formal analysis, Investigation, Data curation, Writing – original draft, Writing – review & editing, Visualization, Project administration. **Lisandra L. Martin:** Conceptualization, Writing – review & editing. **Herre Jelger Risselada:** Conceptualization, Methodology, Software, Formal analysis, Writing – review & editing, Supervision. **Bernd Abel:** Conceptualization, Resources, Writing – review & editing, Supervision, Project administration, Funding acquisition.

## Declaration of Competing Interest

The authors declare that they have no known competing financial interests or personal relationships that could have appeared to influence the work reported in this paper.

## Data Availability

Curvature model for the study of nanoparticle size effects on amyloid fibril stability and molecular dynamics simulation data (Original data) (Zenodo).
